# Importance of Long Non-coding RNAs in the Development and Disease of Skeletal Muscle and Cardiovascular Lineages

**DOI:** 10.3389/fcell.2019.00228

**Published:** 2019-10-18

**Authors:** Sweta Sweta, Tatiana Dudnakova, Smita Sudheer, Andrew H. Baker, Raghu Bhushan

**Affiliations:** ^1^Yenepoya Research Centre, Yenepoya (Deemed to Be University), Mangalore, India; ^2^Centre for Cardiovascular Science, University of Edinburgh, Edinburgh, United Kingdom; ^3^Department of Genomic Science, Central University of Kerala, Kasaragod, India

**Keywords:** non-coding RNA, skeletal muscle, endothelial cell, vascular smooth muscle cell (VSMC), differentiation, mesoderm, myogenesis, cardiovascular diseases

## Abstract

The early mammalian embryo is characterized by the presence of three germ layers-the outer ectoderm, middle mesoderm and inner endoderm. The mesoderm is organized into paraxial, intermediate and lateral plate mesoderm. The musculature, vasculature and heart of the adult body are the major derivatives of mesoderm. Tracing back the developmental process to generate these specialized tissues has sparked much interest in the field of regenerative medicine focusing on generating specialized tissues to treat patients with degenerative diseases. Several Long Non-Coding RNAs (lncRNAs) have been identified as regulators of development, proliferation and differentiation of various tissues of mesodermal origin. A better understanding of lncRNAs that can regulate the development of these tissues will open potential avenues for their therapeutic utility and enhance our knowledge about disease progression and development. In this review, we aim to summarize the functions and mechanisms of lncRNAs regulating the early mesoderm differentiation, development and homeostasis of skeletal muscle and cardiovascular system with an emphasis on their therapeutic potential.

## Introduction

**FIGURE 1:**
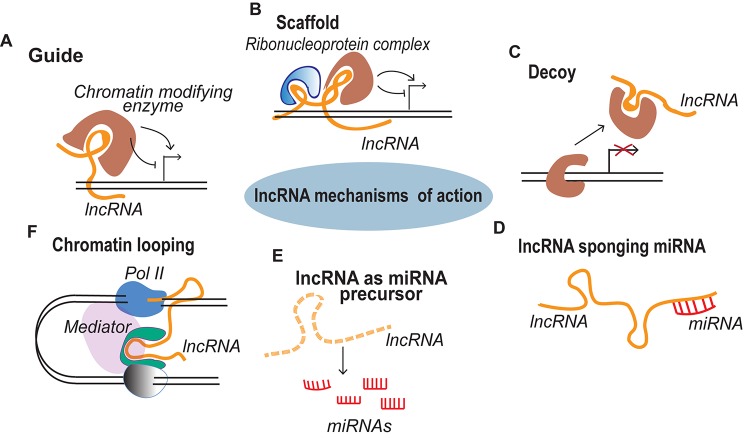
lncRNA mechanisms of action. **(A)** Guide lncRNAs activate or repress gene expression through relocalization of regulatory factors. **(B)** Scaffold lncRNAs aid in the formation of Ribonucleoprotein (RNP) complexes. **(C)** Decoy lncRNAs remove the regulatory factor bound to the genome thereby terminating its regulation. **(D)** lncRNAs sponge the miRNAs, thus inhibiting the miRNA mediated gene repression. **(E)** miRNA precursor lncRNAs function as primary miRNA precursors that are processed into mature miRNAs. **(F)** lncRNA transcription from regulatory regions of the genome initiate long range gene regulation.

Gastrulation results in the formation of the three germ layers - ectoderm, mesoderm and endoderm. The mesoderm is a middle layer between the innermost, endoderm and the outer ectoderm. The transition from epithelial cells to mesenchymal cells marks the formation of mesoderm which is further organized into the paraxial, intermediate and lateral mesoderm (Nakaya and Sheng, 2008; Evseenko et al., 2010). The three parts of the mesoderm are acted upon by several lineage commitment programs and differentiate into the progenitor cells that give rise to musculoskeletal, urogenital and cardiovascular structures of the body (Doss et al., 2012). Cells of these organs have the same genome, but the differences in transcriptionally active and inactive regions of genome guide the precursor cells toward different cell fates (Iwafuchi-Doi and Zaret, 2016). The differences in genomic organization, followed by activation or silencing of genes, are the result of complex gene regulatory networks (GRNs) (Materna and Davidson, 2007). For many years these GRNs were thought to be controlled exclusively by protein coding genes until the discovery of functional non-coding RNA transcripts (ncRNAs) which form an integrated network to shape the cellular environment during different developmental and metabolic processes (Kim and Sung, 2012). These ncRNAs are divided into two categories based on the transcript length—small ncRNAs (<200 nucleotides) and long ncRNAs (>200 nucleotides) (Mercer et al., 2009). Currently, miRNAs are the best-characterized ncRNAs that are well conserved and repress the expression of target mRNA by binding to its 3′ UTR (Majoros and Ohler, 2007). On the other hand, long ncRNAs (lncRNAs) constitute a less characterized but highly diverse class of ncRNAs. lncRNAs are structurally similar to protein-coding genes as most of them are transcribed by RNA polymerase II, 5′ capped and polyadenylated at 3′ end (Bunch et al., 2016). Regardless of their close similarity to the protein-coding mRNAs, lncRNAs lack the potential to code functional proteins. Although there are many lncRNAs that contain putative open reading frames and indeed some have been re-classified to protein-coding genes (Anderson et al., 2015; Nelson et al., 2016; Matsumoto et al., 2017). The number of *bona-fide* lncRNAs identified in human genome is, in general, comparable to that of protein-coding genes, but only a few have been functionally characterized. Functionally, lncRNAs can either act in *cis* by regulating expression of neighboring genes, or in *trans*, regulating the expression of distant genes (Ulitsky and Bartel, 2013). lncRNAs regulate the gene expression by mending the 3-dimentional genome organization, mediating the binding of chromatin modifying proteins or by sequestering the bound regulatory factors or miRNAs by acting as molecular decoys or sponge (Morriss and Cooper, 2017). A summary of different mechanisms of lncRNA mediated genome regulation is represented in Figure 1. A further class of lncRNAs emerging from regulatory regions of the genome such as enhancers can initiate chromatin looping by recruiting chromatin modifying factors to activate or repress transcription at distant genomic location (Wang et al., 2011). In the past decade several research groups have speculated on the functions of lncRNAs in different biological and pathological systems. More specifically, many lncRNAs have been reported to play crucial roles in the development of skeletal muscle and cardiac lineages, and connected diseases. Here, we discuss the current understanding of the roles of lncRNAs in skeletal muscle and cardiac derivatives emphasizing on their therapeutic potential in the associated pathological conditions.

## lncRNAs During Early Mesodermal Specification

**FIGURE 2:**
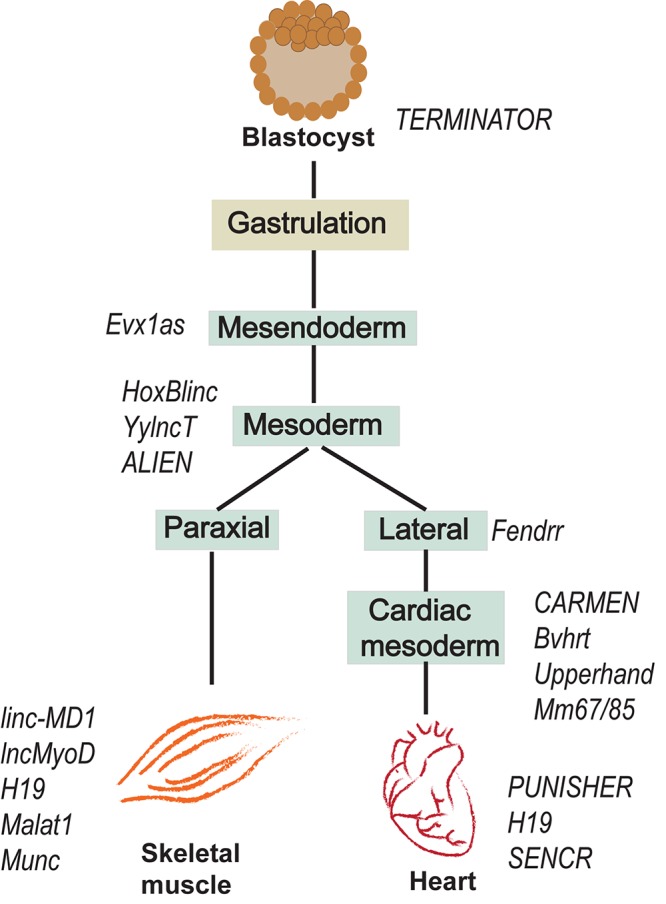
lncRNAs expressed during early development of mesoderm and further differentiation towards skeletal muscle and cardiac lineages.

Recent studies suggest that lncRNAs are important for mesodermal specification and further differentiation, development and function of mesodermal derivatives (Figure 2). For instance, antisense RNA *Evx1as* regulates mesendodermal differentiation toward the mesoderm/posterior streak fate through *cis* regulation of Even-skipped homeobox 1 (Evx1) (Luo et al., 2016). Evx1 is a homeodomain TF that promotes mesoderm differentiation by inhibiting the endoderm/anterior streak gene *GSC* (Kalisz et al., 2012). The expression of the divergent lncRNA *Evx1as* was highly correlated with the expression of *Evx1*. Interestingly, *Evx1as* knockdown exhibited a higher impact on the expression of mesendoderm markers than the knockdown of Evx1, suggesting the possible trans regulation by *Evx1as* independent of Evx1 (Luo et al., 2016). lncRNA *HoxBlinc* is involved in early differentiation and is transcribed from the Homeobox B (Hoxb) locus marking the formation of Flk+ mesoderm with the potential to form hematopoietic and cardiac cells (Deng et al., 2016). As in the case of *Evx1as* and *HoxBlinc*, genomic loci of many other key developmental regulators were found to transcribe divergent lncRNAs, collectively termed as Ying Yang lncRNAs (yylncRNAs). Ying Yang lncRNAs follow tissue-specific expression patterns similar to that of their protein-coding counterparts (Frank et al., 2019). The active locus of the mesoderm specifier BRACHUARY (T) encodes *yylncT* and the expression patterns of the two were nearly identical during mesodermal commitment. The depletion of *yylncT* specifically abolished the differentiation of human embryonic stem cells (hESCs) to mesoderm without affecting the differentiation toward ectoderm and endoderm, emphasizing the mesoderm specific role of *yylncT* (Frank et al., 2019). A summary of lncRNAs regulating early mesodermal differentiation is illustrated in Figure 2.

## lncRNAs Regulating Myoblast Proliferation and Muscle Development

During embryonic development, the paraxial mesoderm develops into segmented somites, organized into ventral sclerotome and dorsal dermomyotome which form the axial skeleton, the skeletal muscle and the dermis of the adult body, respectively (Chal and Pourquié, 2017). Myogenic progenitor cells (MPCs) are formed in the myotome and subsequently become myoblasts that proliferate and differentiate to form myotubes maturing into skeletal muscle fibers. The MPCs are also responsible for the formation of quiescent satellite cells that contribute to regeneration in adult muscles (Chal and Pourquié, 2017). Upon injury, satellite cells in the adult body get activated and become proliferative myoblasts further differentiating to form new muscle fibers. Alterations in regulatory circuitry of myogenesis leads to muscle disorders and diseases such as, hypertrophy and atrophy. This makes it advantageous to identify novel molecular regulators of myogenesis and injury induced regeneration which will aid to discern new therapeutic targets.

Before differentiating into mature myofibers, the myoblasts proliferate with the activation of cell cycle genes. lncRNA *Sirt1AS* is an antisense RNA that promotes myoblast proliferation by protecting Sirt1 mRNA—a suppressor of cell cycle inhibitors—from miR-34a mediated degradation (Wang G.-Q. et al., 2016). *lnc-31* also promotes proliferation by maintaining the expression of critical cell cycle genes, cyclin D1 (Ccnd1), cyclin E (Ccne1) and Cdc25a (Ballarino et al., 2015). *lnc-31* harbors miR-31 precursor sequence, but works independently of miR-31. Despite the poor sequence conservation, both *lnc-31* and its human homologue, *has-lnc-31*, are upregulated during proliferation antagonizing the differentiation process. Furthermore, *lnc-31* and *has-lnc-31* are abundantly expressed in Duchenne muscular dystrophy (DMD) in mice and humans (Ballarino et al., 2015).

**FIGURE 3:**
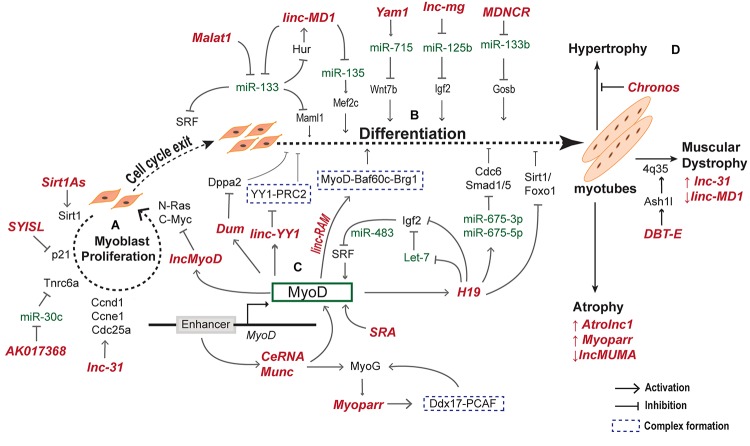
lncRNAs regulate myogenesis at different stages. During myogenesis, myoblast cells proliferate and differentiate into myocytes that fuse together to form multinucleate myotubes. The proliferative and differentiation stages of myogenesis are regulated by several lncRNAs. **(A)** lncRNAs regulating the myoblast proliferation by activating downstream cell cycle genes. **(B)** lncRNAs expression and function during cell cycle exit and differentiation to form myotubes. **(C)** MyoD activated lncRNAs regulate other myogenic factors including MyoD and MyoG to form a complex MyoD–lncRNA–miRNA–mRNA regulatory network during differentiation of myoblasts. **(D)** Examples of lncRNA expressed and regulating various skeletal muscle disorders.

Recently, lncRNA *Syisl* was reported to supress myoblast differentiation, promoting cell proliferation and fusion. *Syisl* escorts the EZH2 of PRC2 to the promoter of cell cycle inhibitor p21 and core myogenic genes like MyoG, Mck, Myh4 (Jin et al., 2018). The same study reported increase in muscle density upon *in vivo* knockout of *Syisl*. lncRNA *Syisl* presents an example of lncRNAs activated in a stage specific manner in regulation of early myogenesis. A schematic representation of lncRNAs regulating the early and late phases of myogenesis with their downstream and upstream effector targets is provided in Figure 3.

## lncRNAs Regulating Myoblast Differentiation

Myoblast differentiation leads to the formation of multinucleate myotubes that form the mature myofiber (Chal and Pourquié, 2017). A number of stage specific factors, such as MyoD, Myf5, MyoG, and MRF4 act in coordination with epigenetic and transcriptional regulators to regulate the myoblast differentiation (Braun and Gautel, 2011). Enhancer regions of MyoD and MyoG were shown to give rise to eRNAs (enhancer RNAs) which in turn regulate their expression. Two such examples are core enhancer eRNA (*^CE^eRNA)* and *MUNC* – both of which are transcribed from the upstream regulatory region of MyoD (Mousavi et al., 2013). *^CE^eRNA* is transcribed from the core enhancer of MyoD regulating its expression in *cis* by facilitating the chromatin accessibility to RNA polymerase ll. Whereas, *MUNC* (also called as *^DRR^RNA*) is transcribed from Distal Regulatory Region (DRR) of MyoD, enhances the expression of MyoD in *cis* and of distantly located myogenic genes, MyoG and Myh3, in *trans* (Mueller et al., 2015). Albeit *MUNC* overlaps the DRR of MyoD, *MUNC* acts on multiple myogenic gene promoters. Similar to eRNAs, the promoter region of mouse and human MyoG transcribes lncRNA *Myoparr* essential for cell cycle withdrawal by activating multiple myogenic factors including the neighboring TF MyoG (Hitachi et al., 2019). *Myoparr* regulates the interaction between MyoD coactivator Ddx17 and histone acetyl transferase and promotes denervation caused atrophy (Hitachi et al., 2019). The discovery of eRNAs and promoter associated RNAs highlight an additional role of regulatory regions of genome, such as enhancers, in genome regulation.

lncRNA and mRNA microarray analysis identified 997 differentially expressed lncRNAs upon MyoD knockdown in C2C12 cells (Guo et al., 2017). Gene ontology predicted that most of these lncRNAs are associated with pathways involved in muscle formation and cell cycle regulation. The study also identified *lncRNA-AK143003* to be significantly regulated by MyoD. In silencing and overexpression experiments *AK143003* acts as differentiation antagonist, but further investigation is required to assess its role and mechanism during myogenesis. This study provides a repertoire of lncRNAs in MyoD network for further validation. Another lncRNA involving MyoD in the regulatory circuitry is *linc-RAM* (Linc-RNA Activator of Myogenesis). It binds to MyoD and facilitates the assemblage of MyoD-Baf60c–Brg1 complex onto the regulatory regions of myogenic genes (Yu et al., 2017). In addition, lncRNA *SRA* acts as a coactivator for master regulator of muscle differentiation, MyoD and *SRA* Knockdown prevented proper expression of muscle genes and differentiation (Caretti et al., 2006). Taken together, these lncRNAs present a second layer of regulation in the MyoD regulatory network. Figure 3C, depicts an overview of MyoD regulated lncRNAs and in turn the lncRNAs modulating MyoD and other myogenic genes.

lncRNAs are known to act as guides and scaffolds by recruiting chromatin or DNA modifying complexes. *DUM* silences Dppa2, an anti-myogenic regulator, by recruiting Dnmts to CpG sites of Dppa2 promoter (Wang L. et al., 2015). *Dum* acts as a promyogenic factor transcriptionally induced by MyoD with highest expression during proliferation and early myogenesis. Ectopic overexpression of *DUM* improved regeneration of muscle mass (Wang L. et al., 2015). *lncMyoD* is another lncRNA activated by MyoD in terminal muscle differentiation suppressing the IGF2-mRNA-binding protein 2 (IMP2) mediated translation of proliferation genes (Gong et al., 2015).

The maternally imprinted lncRNA *H19* is expressed exclusively in embryonic tissues and strongly repressed after birth in all tissue types but skeletal muscles (Brannan et al., 1990; Poirier et al., 1991). *H19* knockdown decreased the differentiation of myoblast cells and mouse satellite cells by derepressing the mRNAs targets of miR-675-3p and miR-675-5p, encoded by *H19*, emphasizing a crucial role of *H19* during skeletal muscle differentiation (Dey et al., 2014). During myoblast differentiation, miR-675-3p downregulated the BMP pathway by targeting anti-differentiation smad1 and smad5, whereas miR-675-5p repressed the DNA replication initiation factor Cdc6 (Dey et al., 2014). Another study suggests that *H19* decrease the expression of myoblast inhibitory genes Sirt1/FoxO1, thus reinforcing the role of *H19* in muscle differentiation (Xu et al., 2017). In addition, *H19* has been shown to act as a molecular sponge for microRNAs belonging to let-7 family, preventing premature myoblast differentiation (Kallen et al., 2013). More recently, a study of double mutant mice lacking MyoD and Igf2 genes elucidates a complex loop of H19 mediated Igf2 repression by MyoD in development of diaphragm muscle (Borensztein et al., 2013). The authors demonstrate that MyoD stabilized the interaction of CS9 mesodermal enhancer with *H19* promoter that accounts for increased expression of *H19* in the presence of MyoD. Furthermore, *H19* represses Igf2 expression in trans (Wilkin et al., 2000). MyoD is in turn negatively regulated due to downregulation of SRF by Igf2 encoded miR-483. Conclusively, H19-Igf2-MyoD are tightly regulated in a negative feedback loop during embryonic myogenesis (Qiao et al., 2011; Borensztein et al., 2013). Overall, it appears that *H19* has a crucial role in myoblast differentiation by mechanistically regulating the key genes such as IGF-2, Sirt1/FoxO1, and microRNAs miR-675 and let-7 during adult as well as embryonic myogenesis.

Similar to *H19*, lncRNA *MALAT1* targets multiple factors during myogenesis. Malat1 is incessantly upregulated during the differentiation of myoblasts to myotubes and its downregulation results in cell cycle arrest in G0/G1 phase, suppressing myoblast proliferation (Watts et al., 2013). A recent study demonstrated a new mechanism of microRNA mediated degradation of *MALAT1* transcripts in myoblast nucleus by miR-181 through Ago2-dependent nuclear RNA-induced silencing complex (RISC) (Chen et al., 2017). *MALAT1* was also found to influence miR-133 mediated SRF targeting during myogenesis (Han et al., 2015). Conclusively, these mechanistic studies provide evidence that a single lncRNA can act at different levels in GRN through multiple of modes of action.

As seen in *H19* and *MALAT1*, sponging of miRNA appears to be a common mechanism of lncRNA action in muscle differentiation. For instance, *lnc-mg* (myogenesis associated lncRNA) promoted Igf2 mediated myogenesis by titrating miR-125b (Zhu et al., 2017). *lnc-mg* was enriched in skeletal muscle and its silencing caused muscle atrophy and loss of muscular endurance during exercise and its overexpression led to hypertrophy. Furthermore, its expression was dynamically induced during differentiation of muscle stem cells (Zhu et al., 2017). Similarly, *linc-MD1* modulates the time of muscle differentiation by favoring the expression of MAML1 and MEF2C by sponging miR-133 and miR-135, respectively (Cesana et al., 2011). In addition to sponging of miR-133, *linc-MD1* is also the host of miR-133 enabling its alternate synthesize from *linc-MD1*, controlled by a feedforward positive loop of HuR protein and *linc-MD1* (Legnini et al., 2014). *linc-MD1* sponges miR-133b derepressing the expression of HuR protein which in turn physically interacts with *linc-MD1* to prevent Drosha cleavage of pri-miR-133 sequence (Legnini et al., 2014).

TF Yin Yang 1 (YY1) is an important regulator of myogenesis that silences multiple genes in myoblasts by recruiting Ezh2 (Enhancer of ZesteHomologue2) (Caretti et al., 2004; Wang et al., 2007). Promoter region of YY1 gives rise to *linc-YY1* exhibiting low expression in proliferating myoblasts, increased at the beginning of the myogenic program with gradual decrease in the late stages of myogenesis (Zhou et al., 2015). Interestingly, YY1 follows a similar expression profile during the process. When myoblasts undergo differentiation, *linc-YY1* is activated by MyoD, which then destabilizes the YY1/PRC2 complex activating pro-differentiation genes (Zhou et al., 2015). Knockdown of *linc-YY1* delayed the expression of many myogenic markers which are direct targets of YY1/PRC2, such as MyoG, MyHC, Tnni2 and a-Actin, and miR-1 and miR-29. This indicates that *linc-YY1* is a pro-myogenic factor whose knockdown in C2C12 cells delayed myogenic differentiation. Genome wide ChIP-Seq in myoblasts revealed that YY1 regulates several lincRNAs in skeletal muscle collectively called as *Yams* (YY1-associated muscle lincRNAs) (Lu et al., 2013). *Yam1* is a lncRNA regulated by YY1 and acts as an anti-myogenic factor and exerts its function in cis through regulation of miR-715 and via targeting Wnt7b expression (Lu et al., 2013). Downregulation of Wnt7b inhibits myogenic differentiation (von Maltzahn et al., 2012). Among other Yams, *Yam-3* also inhibits differentiation while, *Yam-2* and *Yam-4* facilitates early differentiation (Lu et al., 2013). These observations indicate that lncRNAs are not only regulated by key TFs but in turn regulate the function of other TFs. A list of lncRNAs regulating myogenesis is represented in Table 1.

**TABLE 1:** List of lncRNAs regulating skeletal muscle myogenesis and their regulatory mechanisms.

**lncRNA**	**Target**	**Mechanism**	**Function/disease relevance**	**Species**	**References**
AK143003	Unknown	Unknown	Negative regulation of differentiation	Mouse	Guo et al., 2017
AK017368	Tnrc6a	Sponge miR-30c	Promotes myoblast proliferation	Mouse	Liang et al., 2018
Atrolnc1	NF-kb Murf-1	Decoy	Promotes muscle wasting in CKD mice	Mouse	Sun et al., 2018
Charme	Unknown	Unknown	Regulates robustness of skeletal and cardiac myogenesis. *In vivo* depletion in mice resulted in cardiomyopathy	Mouse	Ballarino et al., 2018
Chronos	Bmp7	Unknown	Repressor of skeletal muscle hypertrophy	Mouse	Neppl et al., 2017
^CE^RNA	MyoD	eRNA, regulate pol II occupancy at MyoD	Promotes myogenesis	Mouse	Mousavi et al., 2013
DRR/MUNC	MyoD, MyoG, Myh3	*Cis* and Trans regulation, Pol II recruitment at myogenic promoters	Promotes myogenesis And late stage regeneration	Mouse, Human	Mousavi et al., 2013; Mueller et al., 2015
DBT-E	Ash1l	Guide chromatin remodeling at D4Z4 locus	Expressed in FSHD patients	Human	Cabianca et al., 2012
Dum	Dppa2	*Cis*, recruits Dnmt 1, 3a and 3b at Dppa2 promoter	Promotes differentiation and regeneration	Mouse	Wang L. et al., 2015
H19	Let-7, Igf2, MyoD miR-675, Sirt1/Foxo1	Cis and trans miRNA Sponge, Precursor of miRNA	Promotes myogenic differentiation	Mouse, Human and Cattle	Wilkin et al., 2000; Borensztein et al., 2013; Kallen et al., 2013; Dey et al., 2014; Xu et al., 2017
lnc-31	Cyclins, Cdc25a	*Trans* acting	Maintenance of myoblast proliferation, Upregulated in mdx mice and DMD patient myoblast	Mouse, Human	Ballarino et al., 2015
linc-MD1	miR-133, miR-135, Hur protein	*Cis*, miRNA sponge	Controls time of muscle differentiation, Reduced in DMD patients	Mouse, Human	Cesana et al., 2011
lnc-mg	miR-125b	miRNA sponge	Promotes myogenesis, Knockout and overexpression resulted in atrophy and hypertrophy in mice, respectively	Mouse	Zhu et al., 2017
lncMyoD	IMP2 mediated mRNA translation	Decoy	Terminal muscle differentiation	Mouse, Human	Gong et al., 2015
lncMUMA	miR-762	miRNA sponge	Promotes differentiation, protects against atrophy	Mouse, Human	Zhang et al., 2018
linc-RAM	Myogenic genes	Scaffold, assembly of MyoD-Baf60c-Brg1complex	Promotes differentiation, Impaired muscle regeneration in vivo knockout mice	Mouse	Yu et al., 2017
linc-YY1	PRC2	Trans, Decoy	Promotes differentiation, impaired regeneration upon knockdown	Mouse, Human	Zhou et al., 2015
Malat1	miR-133, Myogenic genes	miRNA sponge, Guide Suv39h1 to myoD binding loci	Promotes myogenesis, Improved regeneration in knockout mice	Mouse, Human	Han et al., 2015; Chen et al., 2017
MDNCR	miR-133b	miRNA sponge	Promote differentiation	Cattle	Li H. et al., 2018
Myoparr	MyoG	Scaffold	Promotes differentiation and muscle	Mouse, Human	Hitachi et al., 2019
Sirt1AS	Sirt1	*Cis*-acting, protects Sirt1 mRNA from miR-34a degradation	Promotes myoblast proliferation	Mouse	Wang G.-Q. et al., 2016
SRA	MyoD	Unknown	Promotes muscle differentiation	Mouse	Caretti et al., 2006
SYISL	P21, myoG, Mck	Guides EZH2 to promoter of target genes	Promotes myoblast proliferation and fusion	Mouse	Jin et al., 2018
Yam 1	Wnt7b	*Cis*-acting, Activates miR-715	Inhibits differentiation	Mouse	Lu et al., 2013

## Therapeutic Potential of lncRNAs in Muscle Regeneration and Diseases

Given the known importance of lncRNAs in skeletal muscle myogenesis, it is not surprising to know that they also regulate the process of muscle regeneration. lncRNAs such as *H19*, *DUM*, *MUNC*, *Yam1* and *lncMyoD* have been shown to regulate regeneration in cardiotoxin (CTX) model of muscle injury (Shiekhattar, 2013; Dey et al., 2014; Gong et al., 2015; Mueller et al., 2015; Wang L. et al., 2015). Upon CTX mediated injury, the expression of *H19* was decreased at days 1–3, followed by increase at days 5–7 and again decreased after the formation of new myofibers at day 14. Interestingly, miR-675-3p and miR-675-5p are co-expressed with *H19* throughout the course of regeneration, making it evident that the pro-differentiation action of *H19* is mediated by microRNAs generated from it (Dey et al., 2014). While *H19* is implicated in regulating regeneration at several stages, expression of *Yam1* appears to be stage specific with highest expression at day 2 followed by a sharp reduction for the rest of the regeneration process (Lu et al., 2013). Likewise, *lncMyoD* is upregulated at days 3–5 and decreased for the remaining regeneration process (Gong et al., 2015). In contrast, *MUNC* regulates late stage muscle regeneration as its knockdown decreased the average diameter of myofiber at day 14 (Gong et al., 2015; Mueller et al., 2015). Among others, the knockout of *linc-RAM* and lncRNA *IRM* in mice displayed impaired muscle regeneration, while the knockdown of *MALAT1* enhanced the regeneration (Chen et al., 2017; Yu et al., 2017; Sui et al., 2019). Paradoxically, lncRNA *LINC00961* encodes a conserved polypeptide, SPAR, downregulated during the regeneration process (Matsumoto et al., 2017). The downregulation of SPAR activates mTORC1 important for skeletal muscle regeneration and hypertrophy. The functional importance of SPAR emphasizes that not all lncRNA-encoded peptides are translational noise and also raises concern regarding the classification of lncRNAs.

Impaired regeneration process leads to conditions like atrophy and hypertrophy characterized by muscle wasting and increase in size of muscle cells, respectively. Cachexia is a condition that involves atrophy and muscle wasting, commonly associated with chronic kidney disease (CKD) (Morley et al., 2006). lncRNA *Atrolnc-1* is elevated in atrophic muscles of mice with cachexia and its inhibition in mice with CKD attenuated muscle wasting (Sun et al., 2018). Targeting *Atrolnc-1* could possibly help ameliorate the severity of CKD. *lncMUMA* was also regulated during atrophy with minimum expression during atrophy development in hindlimb suspension (HLS) mouse model (Zhang et al., 2018). A study on the role of lncRNA in age-associated atrophy identified lncRNA *Chronos* as a repressor of hypertrophic growth through negative regulation of BMP7 (Neppl et al., 2017). The dysregulation of lncRNAs during atrophy and hypertrophy further strengthens their importance in development of muscle fibers.

Similar to atrophy and hypertrophy, lncRNA expression is dysregulated in muscular dystrophy. DMD is the most prevalent type of dystrophy caused by lack of functional dystrophin protein that connects muscle fibers to extracellular matrix (Hoffman et al., 1987). A study using tilling array designed for dystrophin locus identified novel lncRNAs with expression profiles similar to those of dystrophin gene (Bovolenta et al., 2012). These lncRNAs have a repressive role on the full-length dystrophin isoform and their expression is inversely correlated with dystrophin long isoform in the muscle of female dystrophinopathy carriers. Among other lncRNAs, *lnc-31* is upregulated in mdx mice muscle and human DMD patients (Ballarino et al., 2015). On the contrary, *linc-MD1* was reduced in DMD patients (Cesana et al., 2011). Facioscapulohumeral muscular dystrophy (FSHD) is another type of dystrophy characterized by wasting of facial, upper arm and shoulder girdle muscle. In 95% of FSHD cases the defect is a deletion in D4Z4 microsatellite repeat, leading to loss of repressive mark (Wijmenga et al., 1990, 1992). This de-repression is coordinated by a chromatin associated lncRNA *DBT-E* transcribed from D4Z4 repeat through the recruitment of Trithorax group protein at FSHD locus (Cabianca et al., 2012). Although, a few lncRNAs have been reported and studied in muscular dystrophies, they haven’t been studied in non-dystrophic muscle diseases.

Given that lncRNAs are dysregulated in various muscular diseases, they can possibly be novel biomarkers or targets for therapeutic interventions. However, studies investigating lncRNAs in patients with muscular diseases are much languished. Genome wide association studies on different cohorts of muscular disorders may help identify lncRNA loci closely associated with genetic disorders. lncRNAs regulating myogenesis has been well explored in *in vitro* and *in vivo* mouse models, nonetheless their role in humans needs to be investigated in depth.

## lncRNAs in Cardiovascular Development, Proliferation and Differentiation

The heart is the first organ to be formed during mammalian embryogenesis. It consists of a multitude of cell types that are formed through complex lineage commitment programs acting upon lateral plate and paraxial mesoderm (Bruneau, 2013; Stone and Stainier, 2019). The intricate network of signaling pathways and the core transcriptional networks in cardiovascular biology have been extensively investigated for many years. On par with the skeletal muscle development and function, lncRNA discovery has unraveled a new layer of regulation in cardiac biology. A number of studies have identified several lncRNAs crucial for cardiac commitment, differentiation and dysfunction leading to diseased conditions. Many groups have discovered new platforms to identify and catalog lncRNAs that regulate cardiac commitment and pathologies (Grote et al., 2013; Kumarswamy et al., 2014; Ounzain et al., 2014; Viereck et al., 2016). Furthermore, the involvement of lncRNAs as therapeutic targets for cardiovascular diseases (CVDs) is beginning to be understood. In this section, we discuss the role of lncRNAs that specifies and regulates the function of cardiomyocytes, smooth muscle cells and endothelial cells and their roles in CVDs and therapeutics.

## lncRNAs Regulating Early Cardiac Fate and Disease

The minimal regenerative capacity of cardiomyocytes makes it difficult to overcome the damage caused by cardiac diseases. Hence, novel strategies are needed that can improve the regenerative potential of the damaged myocardium. lncRNAs have emerged as novel modulators in cardiac development and regeneration in recent years. *Braveheart (Bvht)* is the first lncRNA identified in mouse cardiac commitment and its depletion in differentiating mouse embryonic stem cells (mESCs) reduced the potential to form cardiac tissue (Klattenhoff et al., 2013). More specifically, *Bvht* functions upstream of Mesp1, a master regulator of cardiac differentiation and depletion of *Bvht* decreased the expression of early cardiac cell surface markers (such as PdgfRa and Flk-1) with consistent increase in hematopoietic markers, suggesting its involvement in regulating cell fate decisions (Klattenhoff et al., 2013). However, the role of *Bvht* in hematopoietic differentiation is unclear and needs further investigation. *Bvht* lacks a human ortholog and this loss in the due course of evolution represents the species-specific differences in heart development.

lncRNA *Fendrr* with its expression restricted to the nascent lateral plate mesoderm, regulates cardiac differentiation by interacting with the PRC complex (Grote et al., 2013). *Fendrr* is imperative for the development of the heart and the body wall. Mechanistically, *Fendrr* binds to two major chromatin modifiers, PRC2 and TrxG/MLL, and recruits these complexes to the promoters of genes involved in inception and differentiation of the lateral plate mesoderm, thus regulating cardiac lineage commitment (Grote et al., 2013).

*CARMEN*, (CAR)diac (M)esoderm (E)nhancer-associated (N)oncoding RNA) is a super enhancer (SE)-associated lncRNA identified in the transcriptome of cardiac precursor cells (CPCs) obtained from the human fetal heart (Ounzain et al., 2014, 2015). The *CARMEN* locus is upstream of two microRNAs known to direct differentiation toward SMC—MiR-143 and -145. *CARMEN* is a conserved lncRNA and shelters a highly active, notch-repressive, SRF/NKX2.5 bound cardiac enhancer, responsible for restricted expression of miR-143 and miR-145 during cardiac development. Notably, the mouse ortholog of *Carmen* and previously mentioned lncRNA *Bvht* are co-located in the mouse genome. Both of the lncRNAs showed maximum expression between cardiac mesoderm and cardiac precursor stages during induced cardiac differentiation of P19CL6 cells, indicative of their involvement in early cardiac specification. While *Bvht* works in *trans*, *CARMEN* functions in *cis* as well as in *trans* and both were found to be essential for maintaining the cardiac identity in neonatal cardiomyocytes (Klattenhoff et al., 2013; Ounzain et al., 2015). Thus, *CARMEN* represents a SE-associated lncRNA that can potentially be manipulated for initiating neocardiogenesis for treating a heart damage.

Global transcriptomic profiling of enhancer transcribed lncRNAs during ESC differentiation into cardiomyocytes in mouse and human reveal co-expression of many of lncRNAs and their predicted downstream targets (Ounzain et al., 2014). The human enhancer transcripts of mm-67, -85, and -130 were significantly upregulated at different time points of cardiac differentiation. More specifically, knockdown of mm-85 derived lncRNA in P19CL6 mouse embryonic carcinoma cells significantly decreased myocardin expression and upregulated mm-67 present within the myocardin gene (Ounzain et al., 2014). This shows the crucial role of mm-85 in regulating myocardin expression in mouse P19CL6 embryonic carcinoma cells.

The growing body of experimental evidence suggests that enhancer derived lncRNAs are important for expression of proximal target genes in cardiac development. lncRNA *Uph* (also named *Upperhand* or *Hand2os1*) is transcribed from upstream enhancer of HAND2, a regulator of heart development and reprogramming of fibroblast to cardiomyocytes (Anderson et al., 2016). Knockout of *Uph* in mouse embryos resulted in the inability of the embryo to develop a right ventricular chamber and the cardiac phenotype emulated by HAND2 knockout embryos (Anderson et al., 2016). More recently, deletion of *Uph* in the mouse upregulated the expression of Hand2 accompanied by the dysregulated cardiac gene program, congenital heart defects and prenatal lethality (Han et al., 2019). While *Uph* is transcribed upstream, lncRNA *Handsdown* (*Hdn*) is located downstream of *Hand2* (Ritter et al., 2019). The genetic analysis in mice demonstrated that *Hdn* is haploinsufficient and *Hdn*-heterozygous mice presented right ventricular hyperplasia with increased levels of *Hand2* (Ritter et al., 2019). Thus, *Uph* and *Hdn* regulate *Hand2* expression in cis thereby playing a crucial role in cardiac development.

A study in human ESCs and zebrafish developmental models identified three lncRNAs implicated at different stages of mesoderm and cardiovascular development, namely, *TERMINATOR*, *PUNISHER* and *ALIEN* (Kurian et al., 2015). These three lncRNAs are conserved from zebrafish to humans and manifest similar stage-specific expression. *TERMINATOR* is essential for early embryonic survival, pluripotency and early mesendodermal differentiation. *TERMINATOR* knockdown in zebrafish resulted in >70% lethal embryos, and developmental arrest and severe cardiovascular defects in the surviving embryos (Kurian et al., 2015). Mesodermal specification is marked by the expression of *ALIEN*. Loss of *ALIEN* resulted in mesoderm-related defects including defective vascular patterning and cardiac chamber formation, alluding toward specific role of *ALIEN* in the early developmental of the progenitor stage common to vascular and cardiomyocyte fates. Silencing of *PUNISHER* was also accompanied by severe impairments in vasculature and cardiac development and function (Kurian et al., 2015). These results were extrapolated to mouse embryos and human ESCs by knockdown of the three lncRNAs followed by microarray hybridization to check for the differential expression of different genes at different stages upon knockdown of these individual lncRNAs (Kurian et al., 2015). Albeit the mechanisms employed by these lncRNAs in controlling the developmental processes remains elusive.

lncRNA *H19* besides its role in early embryonic development, is also critical for late stage cardiac differentiation. During the late stage cardiac differentiation of P19CL6 mouse cells, *H19* knockdown promoted cell proliferation and inhibited apoptosis (Han et al., 2016). *H19* curbs the expression of miR-19b, thereby increasing the expression of miR-19b target Sox-6. Thus, *H19* presents a classic example of lncRNAs having tissue specific roles and targets and provides an explanation for how lncRNAs are capable of regulating a wide variety of cellular processes at different stages in various tissues. Table 2 consolidates the function of lncRNAs involved in cardiovascular development, homeostasis and their relevance to CVDs.

**TABLE 2:** List of lncRNAs with their regulatory mechanisms and physiological impact in cardiovascular biology.

**lncRNA**	**Target**	**Mode of action**	**Function**	**Species**	**References**
ALIEN	Unknown	Unknown	Cardiovascular commitment	Human, Mouse and Zebrafish	Kurian et al., 2015
ANRIL	CDKN2A/B ADIPOR1, VAMP3, C11ORF10	Scaffold	Genetic risk factor for CAD Pro-atherogenic	Human	Bochenek et al., 2013; Holdt et al., 2013
Apf	Atg7	Sponge miR-188-3p	Controls autophagy and MI	Mouse	Wang K. et al., 2015
Bvht	Hand1, Hand2, Mesp1, Nkx2-5, Tbx20	Decoy	Cardiac lineage commitment	Mouse	Klattenhoff et al., 2013
Carl	Pbh2, Bax, Caspase3, Bcl-2	miR-539 sponging	Inhibits mitochondrial fission and apoptosis in cardiomyocyte	Mouse, Human and Rat	Wang et al., 2014b; Li L. et al., 2018
CARMEN	Gata4, mesp1, Nkx2-5, Myh6	*Cis* and *trans* regulation SE-associated	Cardiac specification	Mouse Human	Ounzain et al., 2015
Chast	Plekhm1	*Cis*-regulation	Promotes hypertrophy	Mouse, Human	Viereck et al., 2016
Chaer	Hypertrophy genes	Guides PRC2 to hypertrophic gene loci	Promotes hypertrophy	Mouse, Human and Rat	Wang Z. et al., 2016
Chrf	Myd88	Sponge miR-489	Promote hypertrophy, Elevated in HF tissues	Mouse, Human	Wang et al., 2014a
Fendrr	Foxf1, Gata6, Nkx2-5, Pitx2	Guide PRC2 and TrxG/MLL to promoters of target genes	Cardiovascular development	Mouse, Human and Rat	Grote et al., 2013
Ftx	Bcl2l2	miR-29b-1-3p sponge	Inhibits cardiomyocyte apoptosis	Mouse	Long et al., 2018
GAS5	ANNEXIN A2	Guide	Supress SMC proliferation and migration	Human	Li et al., 2015
GATA6-AS	LOXL2	Decoy	Induced in ECs during hypoxia, involved in EndMT	Human	Neumann et al., 2018
H19	Sox6, MAPK, NF- kB, PTEN, VCAM-1, p21, TGF-β1	Sponge miR-19b, positively regulates miR-675, inhibition of phosphorylation of STAT3.	Proliferation and apoptosis during late stage cardiac differentiation, pro-atherogenic, promotes VSMC proliferation and restenosis, negatively regulates EC aging, prevents glucose induced EndMT	Mouse, Human and Rat	Han et al., 2016; Pan, 2017; Lv J. et al., 2018; Hofmann et al., 2019
MALAT1	TGFBR2/SMAD3, Cyc or CCN A2, B1, B2, Cdk1, ATG7	Sponge miR-145	EndMT, Controls proliferation and migration of ECs	Human	Michalik et al., 2014; Song et al., 2018
LEENE	eNOS	eRNA, recruits Pol II at eNOS promoter	EC homeostasis	Human, Mouse	Miao et al., 2018
MEG3	PTEN, AMPK and JAK-STAT signalling	Sponge miR-21, sponge miR-9	SMC proliferation and migration, supress EC proliferation and angiogenesis	Human	He et al., 2017; Zhu et al., 2018
Mdrl	miR-484	miR-361	Regulates cardiomyocyte mitochondrial fission and apoptosis	Mouse	Wang et al., 2014c
MIAT	VEGF	Sponge miR-93 and miR-150	Pro-hypertrophic	Human, mouse	Zhu et al., 2016; Li Y. et al., 2018
Mm67/77/85/130/132	Unknown	Cis-regulation, eRNAs	Cardiac development and remodelling	Mouse	Ounzain et al., 2014
Mhrt	Brg1	Decoy	Prevents hypertrophy and HF	Mouse, Human	Han et al., 2014
MYOSLID	MYOCD, MRTF-A, TGF-β (SMAD)	Cis and trans-regulation	SMC differentiation and proliferation	Human	Zhao et al., 2016
linc-p21	P53	Mdm2 mediated ubiquitination of p53	Repress VSMC proliferation and induce apoptosis, downregulated in atherosclerotic plaques in mice model	Human, Mouse	Wu et al., 2014
SENCR	MYOCD, *CCL5, CX3CL1, CDH5*	Decoy	SMC contractibility, potentiates mesodermal and endothelial commitment, regulates proliferation and migration of ECs, stabilize EC adherens junction, dysregulation associated with premature CAD and limb ischemia	Human	Bell et al., 2014; Boulberdaa et al., 2016; Lyu et al., 2019
SMILR	HAS2, CENPF	*Cis*-acting	SMC proliferation, Increased expression in unstable atherosclerotic plaque and in plasma of high plasm CRP	Human	Ballantyne et al., 2016; Mahmoud et al., 2019
RNCR3	KLF2	Sponge miR-185-5p	Athero-protective	Human	Shan et al., 2016
ROR	ANP and BNP	Sponge miR-133	Pro-hypertrophic	Mouse, Human	Loewer et al., 2010
PANCR	PITX2	*Cis*-acting	Induced during early differentiation of hESCs to cardiomyocytes	Human	Gore-Panter et al., 2016
PLSR4	Mfn2	Sponge miR-214	Anti-hypertrophic	Mouse	Lv L. et al., 2018
PUNISHER	FOXC1, TAL1	Guide	Endothelial commitment	Human, Mouse and Zebrafish	Kurian et al., 2015
Upperhand	Hand2	*Cis*-acting, eRNA	Heart development	Mouse	Anderson et al., 2016

With the expanding knowledge about the importance of lncRNAs in cell fate decisions and heart development, it is evident that they are operational in maintaining the homeostasis of cardiovascular system. High throughput RNA-sequencing has steered the identification of differentially regulated lncRNAs in cardiac pathologies. One of the common heart conditions is cardiac hypertrophy, characterized by the increase in cardiomyocyte size to compensate for inappropriate cardiac function leading to heart failure (HF). lncRNA *CHAST* was upregulated in murine and human cardiac hypertrophy and *in vivo* depletion of this lncRNA in mouse hypertrophic model prevented and reverted the condition (Viereck et al., 2016). *CHAST* promotes hypertrophy by blocking autophagy and its inhibition prevented HF, thus presenting a promising target for treatment. *Chaer* is another pro-hypertrophic lncRNA which transiently interacts with PRC2 complex and attenuates hypertrophy upon its silencing in pre-stressed heart (Wang Z. et al., 2016). Unlike *CHAST* and *Chaer*, *Mhrt* is a cardioprotective lncRNA regulated by the stress activated Brg1-Hdac-Parp chromatin repressive complex (Han et al., 2014). Pathological stress activates Brg1 leading to aberrant gene expression including inhibition of *Mhrt.* Ultimately, this leads to cardiomyopathy and hypertrophy (Hang et al., 2010; Han et al., 2014). *Mhrt* forms a feedback loop with chromatin remodeling factor Brg1 and its repression results in cardiomyopathy. Restoring the pre-stress levels of *Mhrt* prevented hypertrophy and heart failure. Human ortholog of lncRNA *Mhrt* is repressed in various myopathic hearts demonstrating a conserved lncRNA dependent mechanism in cardiomyopathy (Han et al., 2014). These studies not only highlight the role of lncRNAs in diseases but also emphasize their therapeutic potential.

## lncRNAs Regulating Endothelial Cell Function

**FIGURE 4:**
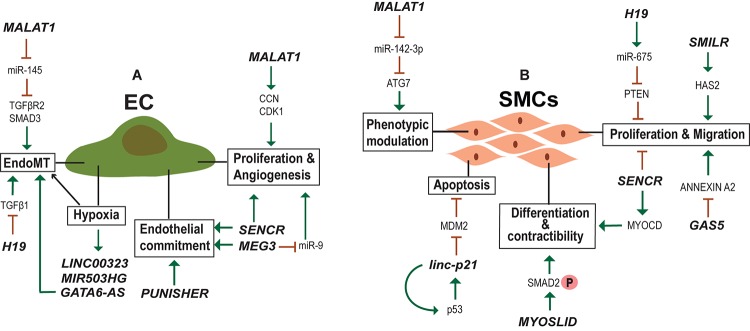
lncRNAs regulating endothelial (EC) and smooth muscle cell (SMC) biology. **(A)** Examples of lncRNA regulating Endothelial cell identity, physiology and activated in hypoxic conditions. **(B)** Examples of lncRNA regulating SMC proliferation, migration, contractibility and phenotypic modulation between contractile and synthetic phenotype.

New blood vessel formation is obviously an important aspect of heart regeneration. In the adult body, perturbed vessel formation may lead to inappropriate blood supply accompanied by shortage of oxygen to the heart resulting in diseased conditions such as myocardial infarction (MI). Endothelial cells (ECs) are important for the formation of the blood vessels and EC dysfunction is one of the early steps in the development of vascular pathologies. Many studies have identified lncRNAs regulating ECs function (Figure 4A) and lncRNAs differentially regulated during vascular diseases. One such example is *MALAT1* which is elevated in TGF-β1-induced endothelial-to-mesenchymal transition (EndMT) where it regulates TGFBR2 and SMAD3 by negatively regulating miR-145 (Xiang et al., 2017). EndMT is a hallmark of various pathological conditions including CVDs, fibrosis and cancer and *MALAT1* regulation of EndMT is a potent target for the development of gene therapy approaches. *MALAT1* is also involved in the regulation of proliferation of ECs. *MALAT1* knockdown increased basal sprouting and migration, inhibiting the proliferation. The reduced proliferation was due to the switch of ECs from a proliferative to promigratory phenotype. In addition, there was a simultaneous increase in the expression of cell cycle inhibitors, such as p21 (Michalik et al., 2014). Furthermore, *Malat1* expression was elevated in hypoxic conditions and its inhibition in a hind limb ischemia mouse model showed reduced capillary density and blood flow recovery (Michalik et al., 2014). Similarly, the inhibition of lncRNA *MANTIS* also prevented the angiogenic sprouting and alignment of ECs subjected to sheer stress (Leisegang et al., 2017). *MANTIS* controls the angiogenesis by activating the ATPase activity of BRG1, facilitating the assemblage of RNA polymerase II onto key endothelial genes like SOX18, SMAD6, and COUP-TFII (Leisegang et al., 2017). Unlike *MALAT1* and *MANTIS*, overexpression of lncRNA *MEG3* suppressed EC proliferation and *in vitro* angiogenesis through negative regulation of miR-9 (He et al., 2017).

Recently, Fiedler et al. (2015) generated an expression atlas of human hypoxia-sensitive lncRNAs with identification of two lncRNAs—*LINC00323* and *MIR503HG*—important for sustaining EC function. The expression levels of growth factor signaling and endothelial TF GATA2 were alleviated upon silencing of these two lncRNAs, accompanied by impaired cell cycle control and blocked capillary formation (Fiedler et al., 2015). Another study aiming to determine hypoxia influence on lncRNA expression in HUVECs identified lncRNA *GATA6-AS* to be upregulated during hypoxia (Neumann et al., 2018). *In vitro* EndMT was reduced upon *GATA6-AS* silencing along with impaired vascular sprouting and endothelial cell migration (Neumann et al., 2018). Additionally, EndMT was regulated by *H19* during diabetic retinopathy where overexpression of *H19* prevented glucose mediated EndMT through regulation of TGF-β1 signaling in a Smad-independent manner (Thomas et al., 2019). Moreover, *H19* was shown to regulate EC aging via negative regulation of age induced inflammatory activation (Hofmann et al., 2019).

lncRNA *SENCR* is important for endothelial cell commitment and function. *SENCR* overexpression substantially enhanced the mesodermal and endothelial commitment of hESC (Boulberdaa et al., 2016). In HUVECs, the upregulation of *SENCR* instigated migration, proliferation and formation of capillary-like structures. Concomitantly, silencing of *SENCR* had reducing effects on these processes. Known migratory and angiogenesis genes were downregulated upon *SENCR* silencing, with no effect on the expression of neighboring *FLI1* gene. Vascular cells derived from patients with limb ischemia and premature coronary artery disease (CAD) showed a reduced level of *SENCR* as compared to control samples (Boulberdaa et al., 2016). Recently, *SENCR* was found to be important for maintaining the membrane integrity of ECs to control the vascular permeability (Lyu et al., 2019). Another important aspect of EC biology is Nitric Oxide synthesis (eNOS) that controls the vasodilation. RNA-seq and chromatin capture study identified eRNA *LEENE* enhancing the eNOS expression by recruiting RNA pol II to the eNOS promoter (Miao et al., 2018). These results suggest the importance of lncRNA in EC homeostasis and endothelial dysfunction which is one of the key triggers of vascular diseases.

## lncRNA in Vascular Smooth Muscle Cell (VSMC) Function

In addition to ECs, VSMC development and function is important in vascular setting. In contrast to terminally differentiated skeletal muscles, VSMCs can undergo reversible phenotypic change between contractile and synthetic phenotypes (Rensen et al., 2007; Davis-Dusenbery et al., 2011). The phenotypic diversity of VSMCs provide the necessary flexibility to the blood vessels to function under different physiological (Figure 4B) and pathological conditions. In normal adult animals, VSMCs exist as highly specialized and differentiated cells with a contractile phenotype. During several vascular pathologies the differentiated VSMCs switch to a proliferative phenotype with surged synthetic activity (Iyemere et al., 2006). Recent studies have identified lncRNA *MALAT1* and a novel lncRNA *SMILR* as important regulators of the switch from a differentiated to a proliferative phenotype of VSMCs (Ballantyne et al., 2016; Song et al., 2018; Mahmoud et al., 2019). Both were shown to promote proliferation and migration of VSMCs, but the mechanism employed and target genes are different for each. *MALAT1* knockdown facilitated the conversion of SMCs from a proliferative phenotype to a differentiated one by inhibiting autophagy (Song et al., 2018). Mechanistically *MALAT1* was found to compete with miR-142-3p to regulate ATG7 mediated activation of autophagy that results in the conversion of VSMCs from a contractile to a proliferative state. While *MALAT1* acts in trans, *SMILR* influences cellular proliferation by regulating the expression of proximal gene HAS2, involved in atherosclerotic lesion formation (Ballantyne et al., 2016). More specifically, *SMILR* affects the late mitotic and cytokinesis phases of cell cycle by interacting with CENPF and this *SMILR*:*CENPF* interaction is in turn regulated by Staufen1 RNA binding proteins (Mahmoud et al., 2019). Alteration of mature VSMC from a contractile phenotype to an osteoblastic phenotype is another major aspect of VSMC biology, which leads to vascular calcification (Iyemere et al., 2006). The transcriptome analysis during calcification of rat VSMCs identified lncRNA *Lrrc75a-as1* as a negative regulator of vascular calcification (Jeong et al., 2019).

Thirty one lncRNAs were identified from RNA-Seq in human coronary artery SMCs (HCASMC), one of which was *SENCR*, that helps maintain the normal SMC differentiated state (Bell et al., 2014). SENCR is transcribed antisense to the Friend Leukemia Integration virus1 (FLI) gene, whereas, *SENCR* knockdown had no effect on the expression of FLI1 or other neighboring genes ruling out *cis*-acting effects of *SENCR* on local gene expression. Attenuation of *SENCR* significantly reduced the expression of many SMC contractile genes, including MYOCD, at both mRNA and protein levels with significant increase in genes inducing motility (Bell et al., 2014). Collectively, these results confirm the regulatory importance of *SENCR* in human coronary artery SMC (HCASMC) differentiation and migration. A study searching for lncRNAs regulated by myocardin (MYOCD/SRF), the master switch for VSMC differentiation, identified lncRNA *MYOSLID* (MYOcardin-induced Smooth muscle Long non-coding RNA, Inducer of Differentiation) as a direct target for MYOCD/SRF and TGFβ/SMAD pathways (Zhao et al., 2016). *MYOSLID* promoted VSMC differentiation and inhibited VSMC proliferation. lncRNAs regulating SMC phenotypic modulation, proliferation and migration might be used as molecular targets in therapies for diseases aggravated by vascular remodeling.

Excessive proliferation of VSMCs is a major attribute of restenosis. *In vitro* overexpression of *H19* accelerated VSMC proliferation by positively regulating miR-675, which in turn downregulates PTEN expression (Lv J. et al., 2018). *H19* and miR-675 were upregulated in injured arterial walls in a rat balloon injury model (Lv J. et al., 2018). lnc-*GAS5* is another lncRNA implicated in proliferation and migration of SMCs. Overexpression of lnc-*GAS5* inhibited the proliferation, migration and reduced apoptosis of human saphenous vein SMCs (HSVSMCs) and conversely, its silencing promoted these cellular behaviors (Li et al., 2015). lnc-*GAS5* function is mediated through a Ca^2+^-dependent RNA binding protein, Annexin A2. Thus, low expression of lnc-*GAS5* increases proliferation and migration of HSVSMCs through AnnexinA2 facilitating in the pathogenesis of the Great Saphenous Veins. *linc-p21* is also shown to regulate SMC proliferation but its expression is downregulated during atherosclerosis (Wu et al., 2014). *linc-p21* silencing in a carotid artery injury mouse model resulted in neonatal hyperplasia due to dysregulation of multiple P53 targets (Wu et al., 2014). The angiotensin II regulated *lnc-Ang362* is a host for miR-221 and miR-222, two known miRNAs implicated in SMC proliferation (Leung et al., 2013). lnc-Ang362, miR-221 and miR-222 were increased in the lung tissues derived from pulmonary arterial hypertension (PAH) patients (Wang et al., 2019). Thus, lnc-Ang362 could be a target for treating PAH.

## Diagnostic Potential of lncRNAs in CVDs

lncRNA with prognostic and diagnostic potential are of particular interest in the clinical arena. A Genome wide association study (GWAS) has identified chromosome 9p21 locus exhibiting the highest association with atherosclerosis (Holdt et al., 2013). This risk locus encodes for lncRNA *ANRIL*, associated with atherosclerotic severity. Further investigations revealed that *ANRIL* functions in *trans*, leading to pro-atherogenic effects such as cell proliferation, increased cell adhesion and decreased apoptosis (Holdt et al., 2013). The association of increased *ANRIL* level with LV dysfunction further highlights its diagnostic and prognostic importance. Similarly, another GWAS study identified that the single nucleotide polymorphism (SNP) associated with MI altered the expression of lncRNA MIAT (Ishii et al., 2006).

Circulating lncRNAs can serve as biomarkers of the diseases making the diagnosis much easier. A transcriptome study identified lncRNA *LIPCAR* in the plasma of patients with heart failure as a novel biomarker which can predict heart remodeling and future death in the patients (Kumarswamy et al., 2014). Another study performed the microarray analysis in the plasma of CAD patients and reported that lncRNA *CoroMarker* as a sensitive biomarker for CAD (Yang et al., 2015). Likewise, circulating *ZFAS1* and *CDR1AS* were found to be decreased and increased in acute myocardial infarction (AMI), respectively (Zhang et al., 2016). Another study reported low levels of lncRNA *GAS5* in the plasma of 30 CAD patients as compared to 30 healthy individuals (Yin et al., 2017).

**FIGURE 5:**
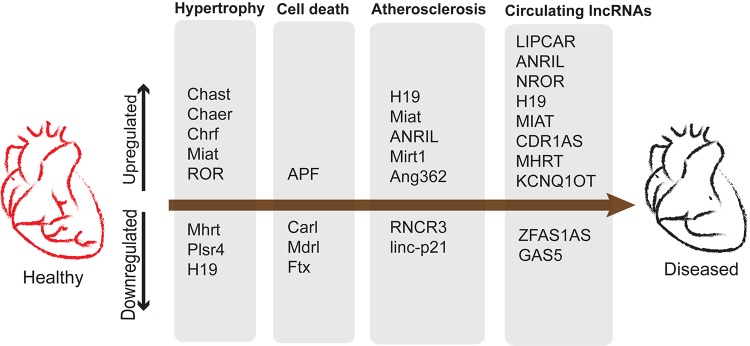
lncRNAs upregulated and downregulated during development of cardiac diseases and potential biomarkers. Cardiac remodeling is characterized by aberrant myocardium growth along with apoptosis and vascular remodeling leading to atherosclerosis.

Promising results for several kinds of lncRNAs were reported similarly (Schlosser et al., 2016). However, the authors observed difficulties in reproducibility of detection. This could be due to the low sensitivity of the conventional lncRNA detection methods. Regardless of the technical challenges the low abundancy of lncRNAs cannot undermine their reported functional importance in many instances highlighted in this review. A summary of lncRNAs as potential biomarkers and their roles in cardiac diseases is represented in Figure 5.

## lncRNA–miRNA Interactions in Skeletal Muscle and Cardiovascular Development and Disease

**FIGURE 6:**
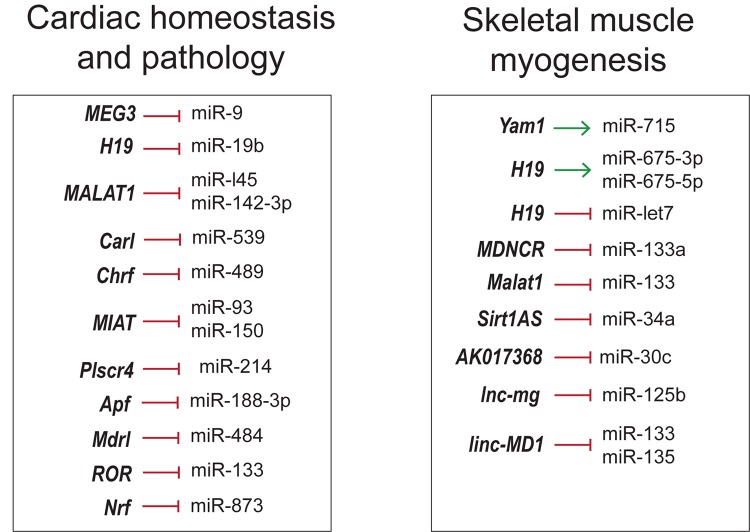
Overview of lncRNA–miRNA interactions in skeletal muscle and cardiovascular development and disease.

The crosstalk between lncRNA–miRNA–mRNA appears to be common in different facets discussed so forth. lncRNAs inhibit the function of miRNA by binding to them in a sequence specific manner, thereby increasing the number of target mRNAs that would otherwise be suppressed by miRNAs. In this regard, comprehensive knowledge of complex lncRNA–miRNA–mRNA-networks would help develop novel RNA-based therapeutics for different diseases (Figure 6).

As seen in skeletal muscle proliferation and differentiation, lncRNA *H1*9, *linc-MD1*, *lnc-mg* and *Malat1* inhibit the action of miRNAs by the sponging mechanism (Cesana et al., 2011; Kallen et al., 2013; Han et al., 2015; Chen et al., 2017; Zhu et al., 2017). However, lncRNAs can regulate the function of miRNAs in many different ways. For instance, lncRNA *Sirt1As* shields the miR-34a binding site on Sirt1 mRNA by binding to the transcripts (Wang G.-Q. et al., 2016). lncRNAs can positively regulate miRNAs by acting as precursor miRNAs as in the case of *H19* giving rise to miR-675-3p and miR-675-5p (Dey et al., 2014).

These RNA networks are not restricted to skeletal muscle but are also noticeable in cardiovascular physiology and disease. *CHRF*, *Plscr4*, *MIAT* and *ROR* were involved in miRNA mediated cardiac hypertrophy regulation. lncRNA *CHRF* triggers cardiomyocyte hypertrophy by sponging antihypertrophic miR-489, which targets Myd88 (Wang et al., 2014a). Similarly, pro-hypertrophic lncRNA *MIAT* regulates anti-hypertrophic miR-93 and miR-150 (Zhu et al., 2016; Li Y. et al., 2018). *ROR* is another hypertrophic inducer that sponges miR-133 (Jiang et al., 2016). In contrast, *Plscr4* is an anti-hypertrophic lncRNA downregulating miR-214 to promote the expression of Mfn2 (Lv L. et al., 2018). Apoptosis and autophagy are among other pathways targeted by competing-endogenous RNAs (ceRNAs). lncRNA *CARL* has been proposed to inhibit cardiomyocyte apoptosis by sequestering miR-539, a microRNA targeting PHB2 (Wang et al., 2014b), a subunit of mitochondrial membrane protein Prohibitin involved in mitochondrial homeostasis (Tatsuta et al., 2005). Cardiomyocyte apoptosis and mitochondrial fission was also inhibited by *Mdrl* via downregulation of miR-361, counteracting the inhibition of miR-484 processing (Wang et al., 2014c). lncRNA *Ftx* inhibited cardiomyocyte apoptosis by preventing miR-29b-1-5p mediated downregulation of Bcl2 (Long et al., 2018). Likewise, *Apf* triggers autophagy by targeting Atg7 through negative regulation of miR-188-3p, an inhibitor of autophagy and myocardial infarction (Wang K. et al., 2015). It is interesting to note that some of the above mentioned networks are well conserved across species, suggesting the crucial roles, they have in cardiovascular biology. Hence, modulating lncRNA-miRNA-mRNA pathways could possibly be a novel strategy to suppress cardiomyocyte loss in order to protect against myocardial infarction (MI) and tailor therapies for hypertrophy. Thorough understanding of these networks in development and disease could help design better therapeutic strategies in the field of regenerative medicine.

## lncRNA Therapeutics and Challenges

There has been an increased interest in the understanding of lncRNA regulation in disease systems due to their relatively restricted expression patterns and different possible actions on cellular function(s). Given that several of these ncRNAs are dysregulated in disease conditions, modulating the levels of such lncRNAs appears to be a promising approach for therapeutics and preclinical testing. However, only a few lncRNAs have been studied in depth in relevant *ex vivo* and *in vivo* systems. Upregulation or downregulation/inhibition of lncRNA function has been the most rigorously adopted methods to understand their therapeutic potential in muscular defects and cardiovascular damage. The most commonly used gene delivery methods for RNA based therapeutics are recombinant viral systems such as adenovirus, lentivirus and adeno-associated viruses (AAVs), which are employed either to inhibit or to overexpress the mRNA, miRNA, lncRNA whole transcripts (Eulalio et al., 2012; Gomes et al., 2017; Gabisonia et al., 2019). The use of RNAi and antisense oligonucleotides (ASOs) present the most commonly used approaches for selective downregulation of potential lncRNAs. On the contrary, the upregulation of lncRNAs can be achieved by viral vectors that are very efficient for muscle delivery. Using the above mentioned strategies, there have been promising results toward miRNA targeting and protein coding transcripts in preclinical systems, which reached clinical trials. However, using the same delivery methods the therapeutic relevance of lncRNAs remain to be resolved.

The siRNA mediated pharmacological inhibition of cytoplasmic lncRNA *SMILR* in the *ex vivo* vein graft model significantly reduced the SMC proliferation to ameliorate the effects of vascular remodeling (Mahmoud et al., 2019). In another approach using nanoparticle mediated transfection of siRNA against lncRNA *Chaer* directly into mouse heart decreased the cardiac hypertrophy and fibrosis and restored cardiac function (Wang Z. et al., 2016). The inhibition level achieved by shRNAs and siRNAs are, in general, limited to the cytoplasmic lncRNA molecules. Hence, on the other hand, ASOs or GapmeRs are more suitable for nuclear lncRNAs that direct Dicer independent degradation of the target lncRNA by RNase H activity (Fluiter et al., 2009; Bennett and Swayze, 2010; Lennox and Behlke, 2016). The ASO technology has fewer off target effects than the small RNA mediated approach. One such example is GapmeR mediated inhibition of lncRNA *Chast* for the prevention and regression of cardiac hypertrophy *in vivo* (Viereck et al., 2016). No noticeable side effects of GapmeR treatment were reported. In another study, GapmeR mediated knockdown of *Malat1* in mice muscle resulted in poor blood flow recovery and diminished capillary density (Michalik et al., 2014). In addition, *in vivo* therapeutic intervention using GapmeRs targeting lncRNA *Wisper* suppressed cardiac fibrosis and improved function (Micheletti et al., 2017). So far, none of the antisense based lncRNA drugs have been tested in clinical studies but, this strategy has been proven to be effective and safe in a clinical trial targeting liver miR-122 with LNA-based antimiRs (mirvarsen) (Zumla et al., 2013). The newer generation of ASOs provide spatial control for target delivery and with further improvements, this could be translated for lncRNA inhibition in future clinical trials.

In comparison to inhibition, overexpression of cardioprotective lncRNAs appear to be much more challenging attributing to their length and locus complexity, and sometimes incomplete knowledge of the complete sequence identity and isoform structure in the current genome annotation. In this context, the major challenge is efficient transportation of these large transcripts across the membrane barriers and determining the potential toxicity. The transgenic gene activation of the cardioprotective lncRNA *Mhrt* demonstrates the first overexpression study highlighting the translational importance of delivery of lncRNA as a drug (Han et al., 2014). In another example, *Chast* overexpression using AAV9 (adeno-associated-virus serotype 9) viral particles promoted cardiac hypertrophy in mouse heart, suggesting it to be a crucial target for cardiac hypertrophy (Viereck et al., 2016).

**FIGURE 7:**
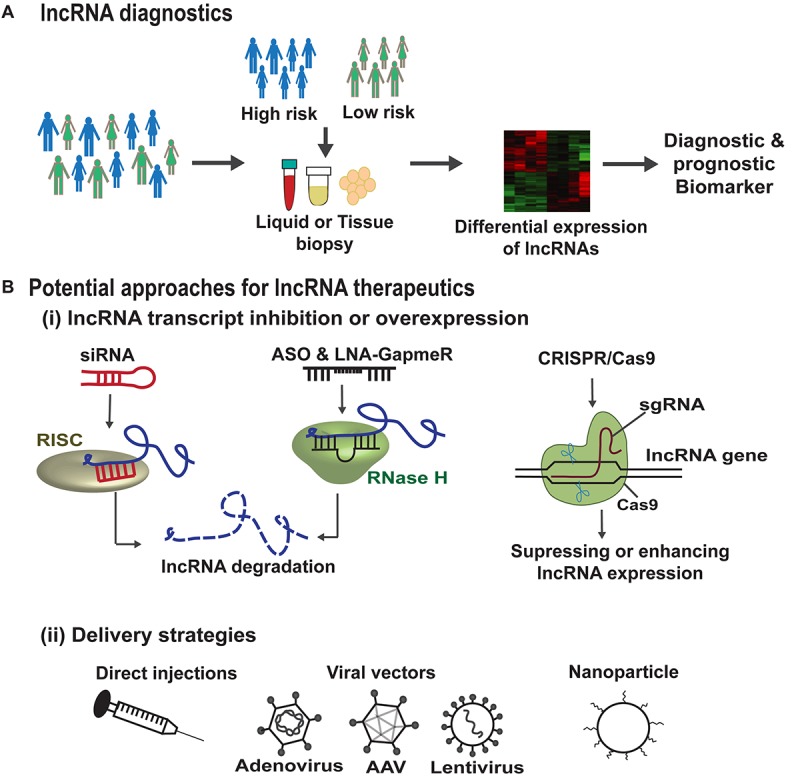
lncRNA based diagnostics and therapeutic strategies. **(A)** Genome wide association studies (GWAS) help in the identification of lncRNA single nucleotide polymorphisms (SNPs) associated with disease susceptibility. These SNPs can alter the lncRNA expression in the body fluids or tissues which can serve as diagnostic or prognostic markers. In addition, improvements in RNA-sequencing technologies can lead to the identification of lncRNAs as biomarkers/disease targets in different diseases. **(B)** (i) Summarizes the potential approaches for lncRNA therapeutics, such as siRNAs that are associated with RNA-induced silencing complex (RISC) bind and cleave the target lncRNA. ASOs and LNA-GapmeRs bind to the target lncRNA in sequence specific manner leading to RNase H mediated lncRNA degradation. CRISPR/Cas9 gene editing tool can make deletion or insertion in the DNA sequence of the target lncRNA to either enhance or abrogate its expression. **(B)** (ii) Demonstrates the different lncRNA delivery strategies such as direct injections, viral particles, or via encapsulation in nanoparticles.

Advancements in RNA-sequencing technologies have led to the identification of several lncRNAs in different diseases suggesting them to be crucial targets for therapeutics. In addition, due to their distinct expression patterns they can be powerful tools for diagnostics, personalized medicine and drug development. A brief description of lncRNA-based diagnostic and therapeutic strategies is depicted in Figure 7. There are several key challenges in any lncRNA-focused therapy. Firstly, the lncRNAs due to their pleiotropic actions regulate multiple targets and hence may not be target-specific and can act via more than one mechanism in a diseased state. In addition, low conservation of lncRNAs across species, is another challenge, for example a human specific lncRNA lacking conservation in mouse, makes its preclinical testing inappropriate (Lu and Thum, 2019). A further challenge in lncRNA therapeutics is the fact that lncRNAs are often integrated in complexes which make them inaccessible for access. Small molecules such as aptamers may help the lncRNA binding with the interacting protein complexes or induce conformational changes in the secondary or tertiary structures of lncRNA (Lunse et al., 2010). This is mainly important, where the lncRNA expression does not attribute to the disease phenotype but, the lncRNA interaction with other molecules accounts for the disease. Hence, the secondary and tertiary structure of lncRNAs and their structure-function relationship should to be resolved before lncRNAs enter into therapeutics.

The other translational limitations of lncRNA therapeutics is the lack of efficient and safe delivery systems for a controlled and targeted release. The delivery vehicle should ensure high transfection efficiency with minimal cytotoxicity but also controlled release of the lncRNA based drug during the complete process. lncRNA levels can be manipulated using viral vectors, however, the use of viral delivery methods is restricted due to the associated issues including off-target effects, activation of host immune response and the risk of insertional mutagenesis, although clinical evidence in gene therapy to date demonstrates a favorable situation. To overcome these risks, researchers believe non-viral vectors with chemical modifications and/or nanoparticles targeted to specific cell type would be advantageous in clinical trials, however, are relatively inefficient dampening down the possibility for clear efficacy signals. Finally, the use of gene editing technique such as CRISPR/Cas9 has provided both loss- or gain-of-function of lncRNA in *in vitro* and *in vivo* studies (Aparicio-Prat et al., 2015; Leisegang et al., 2017; Ballarino et al., 2018). Although, similar delivery issues need to be addressed with the use of gene editing tools *in vivo* too.

## Conclusion

Several lncRNAs have been identified to be involved in development and pathophysiology by regulating gene expression at DNA, RNA and protein levels. Their specific expression during differentiation and disease helps qualify them as key regulators and potential therapeutic targets. However, at present the use of lncRNAs in therapeutics is in its inception. There are still certain technical issues that need to be addressed, such as the difficulties in targeting, accurate outcome predictions and discrepancies in knockdown phenotypes in *in vitro* and *in vivo*. Further improvement in detection, silencing approaches, mechanistic understanding and development of *in vivo* models would uncover the full potential of lncRNAs as diagnostic and possibly therapeutic targets.

## Author Contributions

SwS collected and analyzed the data, prepared the figures, and wrote the manuscript. TD and SmS corrected and gave suggestions to improve the manuscript. RB conceived, planned, analyzed, wrote, coordinated, and finalized the manuscript. AB wrote, reviewed, coordinated, and finalized the manuscript.

## Conflict of Interest

The authors declare that the research was conducted in the absence of any commercial or financial relationships that could be construed as a potential conflict of interest.
